# A mechanistic assessment of the relationship between gut morphology and endozoochorous seed dispersal by waterfowl

**DOI:** 10.1002/ece3.4544

**Published:** 2018-10-30

**Authors:** Erik Kleyheeg, Bart A. Nolet, Sandra Otero‐Ojea, Merel B. Soons

**Affiliations:** ^1^ Ecology and Biodiversity Group, Institute of Environmental Biology Utrecht University Utrecht The Netherlands; ^2^ Department of Animal Ecology Netherlands Institute of Ecology (NIOO‐KNAW) Wageningen The Netherlands; ^3^ Theoretical and Computational Ecology IBED, University of Amsterdam Amsterdam The Netherlands

**Keywords:** digestive tract, endozoochory, mallard, seed size, water birds, wetland ecology

## Abstract

Many plants and invertebrates rely on internal transport by animals for long‐distance dispersal. Their dispersal capacity is greatly influenced by interactions with the animal's digestive tract. Omnivorous birds adjust their digestive tract morphology to seasonally variable diets. We performed feeding trials in waterfowl to unravel how changing organ size, in combination with seed size, affects dispersal potential. We subjected captive mallards to mimics of summer (animal‐based), winter (plant‐based), and intermediate diets, and analyzed gut passage of seeds before and after the treatment (trials 1 and 2). To test the effect of gut morphology on seed digestion, we measured digestive organ size after euthanasia. Three hours before euthanasia, differently sized seeds were fed to test how seed size affects gut passage by determining their relative position in the digestive tract (trial 3). Trials 1 and 2 showed that intact seed passage was lower in the plant‐based than in the animal‐based diet group. Retention time changed only within groups, decreasing in the animal‐based, and increasing in the plant‐based diet group. No post‐diet differences in organ size were detected, probably due to large between‐individual variation within groups. Digestive tract measures did not explain variation in seed survival or retention time. Trial 3 revealed that small seeds pass the digestive tract rapidly, while large seeds are retained longer, particularly in the gizzard. Differential retention in the gizzard, the section where seeds can be destroyed, is likely why larger seeds have a lower probability to pass the digestive tract intact. Our results confirm that rapid, flexible adaptation to diet shifts affects seed digestion in waterfowl, although we could not conclusively relate this to organ size. Large interindividual variation in digestive efficiency between mallards feeding on the same diet may provide opportunities for seed dispersal in the field throughout the annual cycle.

## 1 INTRODUCTION

Animals like the mainly omnivorous dabbling ducks play an important role in the long‐distance dispersal of many plant and invertebrate species, in particular through internal transport following ingestion (Brochet, Guillemain, Fritz, Gauthier‐Clerc, & Green, 2009; Figuerola & Green, 2002; Viana, Santamaría, Michot, & Figuerola, 2013). Gut passage survival and retention time are two fundamental components of so‐called endozoochorous dispersal (Leeuwen, Velde, Groenendael, & Klaassen, 2012; Schupp, Jordano, & Gómez, 2010; Will & Tackenberg, 2008). Together, they determine the potential dispersal distance, although this also depends heavily on the spatial behavior of the disperser animal (Figuerola & Green, 2002; Kleyheeg, 2015; Kleyheeg, Treep, Jager, Nolet, & Soons, 2017; Will & Tackenberg, 2008). Endozoochorous dispersal is crucial to the population dynamics of numerous plant species in a wide range of ecosystems (e.g., Pakeman, 2001; Jordano, García, Godoy, & García‐Castaño, 2007; Sasal & Morales, 2013; Lovas‐Kiss, Vizi, Vincze, Molnár, & Green, 2018) and has the advantage over other dispersal mechanisms that it is often directed toward habitat patches that are suitable for establishment and not necessarily physically connected (Howe & Murray, 1986; Kleyheeg et al., 2017; Wenny, 2001). A broad range of aquatic and terrestrial plant species benefit from this by dispersing via the guts of waterfowl (Figuerola and Green, (2002); Leeuwen, Velde, et al., 2012; Lovas‐Kiss et al., 2018; Soons, Brochet, Kleyheeg, & Green, 2016; Kleyheeg, Klaassen, & Soons, 2016; Farmer, Webb, Pierce, & Bradley, 2017). Recent mechanistic models predicting dispersal patterns shaped by migrating waterfowl (Soons, Vlugt, Lith, Heil, & Klaassen, 2008; Viana et al., 2013 ; Viana, Santamaría, Michot, & Figuerola, 2013) and resident waterfowl (Kleyheeg et al., 2017) have highlighted the importance of variation in seed gut passage survival and retention time for the outcome of dispersal events.

Experimental feeding trials with captive waterfowl have repeatedly shown that seed survival and retention time depend primarily on seed size (e.g., Soons et al., 2008; Mueller & Valk, 2002; Wongsriphuek, Dugger, & Bartuszevige, 2008; Reynolds & Cumming, 2016) and digestive tract performance (e.g., Figuerola, Green, Black, & Okamura, 2004; Leeuwen, Tollenaar, & Klaassen, 2012; Kleyheeg, Leeuwen, Morison, Nolet, & Soons, 2015). The size or volume of seeds consumed by waterfowl varies over several orders of magnitude (Soons et al., 2016) and is an important determinant of endozoochorous dispersal capacity between and within plant species (Figuerola, Charalambidou, Santamaría, & Green, 2010; Soons et al., 2008). The negative relation between seed size and intact gut passage is thought to be related to longer retention of larger seeds in the gizzard (Kleyheeg et al., 2015; Soons et al., 2008), although some studies found no delayed gut passage of large seeds (Figuerola et al., 2010; Traveset, 1998). The mechanism underlying seed size‐dependent gut retention times in waterfowl has never been assessed experimentally.

Similarly, digestive performance is highly variable between waterfowl species (Barnes & Thomas, 1987; Kehoe & Ankney, 1985), between individuals of the same species (Kleyheeg et al., 2016; Whyte & Bolen, 1985), and even within individuals over time (Charalambidou, Santamaría, Jansen, & Nolet, 2005; Kleyheeg et al., 2015; Leeuwen, Tollenaar, et al., 2012). Digestive tract morphology of waterfowl is highly adaptive, as shown for small intestine length, which increased dramatically with increasing food consumption in Bewick's swans (*Cygnus bewickii* Gils et al., 2008) and mallards (*Anas platyrhynchos* Miller, 1975), as well as for gizzard size, which responds rapidly to changes in diet quality in the field (Kleyheeg et al., 2016; Whyte & Bolen, 1985) and in captivity (Kehoe, Ankney, & Alisauskas, 1988; Miller, 1975). For example, monthly mean mallard gizzard size and small intestine length varied by 19% and 41%, respectively, over the course of the nonbreeding season in the Netherlands (Kleyheeg et al., 2016). Experiments with captive mallards demonstrated that gizzard size and small intestine length increased within 10 days when switching from an animal‐based (low‐fiber) to a plant‐based (high‐fiber) diet (Kehoe et al., 1988; Miller, 1975), with potential effects on the efficiency of digestion of ingested plant seeds and other small organisms (Charalambidou et al., 2005; Figuerola et al., 2004). Theory predicts that this mechanism enables birds to switch seasonally between different food types without sacrificing assimilation efficiency per unit time (Leeuwen, Tollenaar, et al., 2012), while maintaining a constant retention time (Gils et al., 2008). Charalambidou et al. (2005), however, found that plastic markers were retained longer in mallards on a plant‐based diet than in those on an animal‐based diet. Similarly ambiguous is the direct effect of digestive tract morphology on seed digestion, for which field studies found contradicting results (Figuerola et al., 2004; Kleyheeg et al., 2016). A direct link between the efficiency of seed digestion and waterfowl digestive tract morphology has never been shown.

Here, we rigorously tested this theoretical framework to understand how variation in digestive tract morphology affects to potential of waterfowl species to disperse plant species. We experimentally investigated the flexibility of mallards in adapting the size of their digestive organs to changes in diet quality, and how this flexibility translates into changes in gut passage and dispersal potential of plant seeds. Specifically, we performed seed feeding experiments with mallards before and after adaptation to an animal‐based, plant‐based, and mixed diet, and concluded these experiments by quantification of their digestive tract morphology. To evaluate the extent to which seed size modulates variation in retention time, we fed the mallards differently sized seeds shortly before euthanasia and analyzed the position of the seeds in their digestive tracts. We hypothesized that digestive organ size would increase with the proportion of plant material in the diet, with a direct negative effect of gizzard size on seed survival, but without a change in retention time. Additionally, we expected that seed size‐dependent variation in retention time is mostly determined by differential retention in the gizzard, which could explain why large seeds generally have a lower gut passage survival.

## 2 MATERIALS AND METHODS

### 2.1 Study species

**Figure 1:**
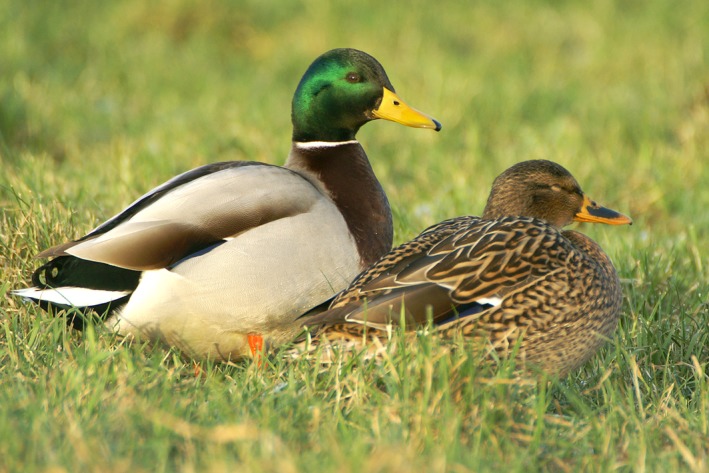
The mallard (*Anas platyrhynchos*) is among the most abundant waterbird species in the Northern Hemisphere and an important disperser of plant and animal propagules

In this experiment, we used 18 captive mallards (11 females and seven males; Figure 1) that were housed at the Netherlands Institute of Ecology (NIOO‐KNAW, Wageningen, the Netherlands). The birds were all born in captivity and at least three years of age. Before the experiment, all mallards were kept on a mixed diet of grains and commercial waterfowl pellets (Anseres 3^®^, Kasper Faunafood, Waalwijk, the Netherlands) in an outdoor facility. We used mallards because of their unselective and seasonally variable foraging behavior (DuBowy, 1988; Hoyo, Elliott, & Sargatal, 1992), their high abundance in most of the Northern Hemisphere (Hoyo et al., 1992), and the high adaptive plasticity of their digestive tract morphology in response to diet changes (Gils et al., 2008; Heitmeyer, 1988; Kehoe et al., 1988; Kleyheeg et al., 2016; Miller, 1975).

### 2.2 Diet adaptation

We randomly divided the mallards into three diet groups (Table 1) and kept the groups in separate compartments in an indoor waterbird facility for the duration of the experiment. We used a plant‐based diet consisting of a mixture of wheat and corn grains (typically characterized by high carbohydrate and fiber content), a more easily digestible animal‐based diet of trout pellets (F‐1P Optiline, Milkivit, Burgheim, Germany), and an intermediate diet composed of a 1:1 mixture of the plant‐based and animal‐based diets. These diets mimicked the fiber contents of natural winter, summer and autumn/spring diets of mallards and other dabbling ducks (Dessborn et al., 2011; DuBowy, 1988). The intermediate diet most closely resembled the relatively balanced commercial diet of the mallards before the experiment. The mallards were first kept on their experimental diet for four weeks to make sure that they had enough time to adjust to their new diet. According to Kehoe et al. (1988), the strongest changes in digestive tract morphology occur within 10 days following a diet shift. Food and water were available ad libitum at all times. The birds were checked every day and weighed at least once every three days.

**Table 1:** Diet, composition, and mean body mass of mallards in the experimental groups

Diet type	Diet composition	Group composition	Body mass (g) (± *SD*)
Animal‐based	Trout pellets	4 ♀ + 2 ♂	1,150 (± 184)
Intermediate	1:1 mixture	4 ♀ + 2 ♂	1,130 (± 88)
Plant‐based	Mixed grains	3 ♀ + 3 ♂	1,087 (± 143)

### 2.3 Trial 1 and 2: Effects of diet type on seed gut passage

To analyze how adaptation of mallards to diet quality affects their seed dispersal capacity, we tested their digestive performance using 24‐hr feeding trials directly before (trial 1) and after (trial 2) the diet adaptation period. At the start of both feeding trials, each mallard was force fed with feeding pellets containing 200 seeds of four plant species: *Berula erecta*,* Comarum palustre*,* Lysimachia vulgaris,* and *Mentha aquatica* (Table 2), adding up to a total of 800 seeds per individual per trial. These species were representative in size and shape of seeds most often consumed by waterfowl (Soons et al., 2016) and survived gut passage relatively well in earlier feeding experiments (Kleyheeg et al., 2015; Soons et al., 2008). The 3‐ to 4‐cm‐long pill‐shaped feeding pellets were made of crushed commercial waterfowl pellets, which were wetted to create a dough‐like substance, similar to the pellets used successfully in previous feeding trials (Kleyheeg et al., 2015; Leeuwen, Tollenaar, et al., 2012). The feeding pellets were made one day before the feeding trials and kept overnight in a refrigerator at 4°C.

**Table 2:** Seed species, volumes, and numbers fed to individual mallards in trials 1, 2, and 3

Plant species	Seed volume (mm^3^)	Trial	*N* seeds fed
*Berula erecta*	0.80	1, 2	200
*Comarum palustre*	0.81	1, 2	200
*Lysimachia vulgaris*	0.65	1, 2	200
*Mentha aquatica*	0.06	1, 2	200
*Bolboschoenus maritimus*	3.70	3	50
*Carex pseudocyperus*	0.81	3	50
*Epilobium palustre*	0.07	3	50
*Hypericum tetrapterum*	0.05	3	50
*Iris pseudacorus*	153.77	3	25
*Lycopus europaeus*	0.37	3	50
*Persicaria pensylvanicum*	2.93	3	50
*Sparganium erectum*	19.15	3	25

After force feeding, the mallards were individually placed in wooden cages (0.6 × 0.5 × 0.5 m) with mesh wire front, back, and floor. The birds were unable to see each other, but could still hear each other. A plastic tray was placed underneath each cage to catch the feces that fell through the mesh wire floors. Feces were collected from the trays every hour for 12 hr and once more after 24 hr. During the feeding trials, the mallards were deprived of food, but had ad libitum access to water.

Feces were subsequently sieved on a 100‐μm mesh after which seeds were collected from the residue and counted under a dissecting microscope with 10–40 × magnification. Total retrieval of intact seeds (seeds that appeared undamaged and potentially viable) was calculated as the fraction of all seeds per species that were retrieved over the 24‐hr feeding trials per individual mallard. Retention time was calculated as the weighted mean time between feeding and collection of seeds per species. For this calculation, we used only the high‐resolution data, that is, seeds retrieved hourly during the first 12 hr (comprising 90% of all seeds retrieved in this study).

### 2.4 Trial 3: Seed size effect and digestive tract analysis

The aim of the third feeding trial was to further disentangle the mechanisms behind seed size‐dependent variation in retention times, by assessing the patterns in digestive tract passage of differently sized seeds. To this end, the mallards were subjected to another feeding trial. In this trial 3, starting directly after the end of trial 2, the mallards were force fed with a total of 350 seeds of eight plant species, greatly differing in size, with mean seed volume ranging from 0.05 to 153.77 mm^3^ (Table 2; volumes based on the LEDA Traitbase (Kleyer et al., 2008)). These species belonged to different genera to prevent confounding phylogenetic effects, were representative for the wide range of seeds consumed by mallards, and were also used in earlier experiments (Kleyheeg et al., 2016; Soons et al., 2016, 2008 ). They were selected to be relatively similar in shape, that is, not very elongated or flat, to avoid confounding effects of seed morphology other than size on digestive tract passage. Only plain seeds without appendages were fed. We used different species than in trials 1 and 2 to avoid contamination from seeds that might still be retained in the digestive tract. The conditions during trial 3 were kept the same as during the previous two trials, except that the trays underneath the cages were emptied only once, at the end of the trial. Based on the results of earlier feeding trials, we expected retrieval to peak after approximately 3 hr (Kleyheeg et al., 2015; Leeuwen, Velde, et al., 2012), indicating that seeds have spread across the entire digestive tract by this time. Therefore, exactly three hours after force feeding, the mallards were euthanized and the position of the seeds in the digestive tracts was determined.

For euthanasia, the mallards were first sedated by inhalation of isoflurane and subsequently injected with a lethal dose of t61 in a vein in the leg. Directly afterwards, the total body length of the mallards was measured from the tip of the bill to the tip of the tail and their complete digestive tracts were removed. The digestive tracts were separated in seven parts: esophagus, proventriculus, gizzard, the first and second half of the small intestine, ceca, and colon. Their contents, as well as the collected feces, were sieved following the same procedure as in trial 1 and 2. We expressed the number of seeds per species found in the separate organs and in the feces as a proportion of the number ingested. For each digestive tract part, we measured length (before and after emptying) to the nearest 0.5 cm, and fresh weight (empty) to the nearest 0.01 g. Additionally, we measured the gizzard size by volumetric displacement of water in a measuring cylinder to the nearest mL. This study was carried out under license number NIOO13.13 of the animal experiments committee of the Royal Netherlands Academy of Arts and Sciences (DEC‐KNAW).

### 2.5 Data analysis

First, we tested whether differences existed between the diet groups in the intact retrieval of seeds both before (trial 1) and after (trial 2) the diet treatment (test 1 as presented in Table 3). For this, we used generalized linear mixed‐effects models (GLMMs) with binomial error distribution and logit link function, including the proportion of retrieved intact seeds as dependent variable, diet treatment as fixed factor and seed species and mallard ID as random effects to control for seed species effects and repeated measures within individuals. Secondly, to formally test the interaction effect between diet treatment and feeding trial (i.e., before and after treatment), we ran the same GLMM with feeding trial number (first or second) and the interaction between diet treatment and feeding trial number as additional fixed effects (test 2). We used backward selection of full models to find the best fitting model, and subsequently used likelihood ratio tests between the models with and without the terms of interest to test their respective contributions to the model. Significant diet effects were further explored using Tukey's HSD post hoc tests. The same procedure was followed for testing the effect of diet treatment on mean retention time, but we used linear mixed‐effects models (LMMs) with normal error distribution, with the same fixed and random effects (tests 3 and 4).

**Table 3:** Summary of test results (chi‐square statistic, degrees of freedom, and *p*‐value) of all tests as described in the methods section

				*χ* ^2^	*df*	*p*
Trial 1 and 2						
1.	Feeding trial 1		RTR ~**DIET** + (1|SP) + (1|ID)	7.3	2	0.026
	Feeding trial 2		RTR ~**DIET** + (1|SP) + (1|ID)	6.7	2	0.035
2.	RTR ~DIET + FT +**DIET:FT** + (1|SP) + (1|ID)			154.8	2	<0.001
	post hoc plant based		RTR ~**FT** + (1|SP) + (1|ID)	5.4	1	0.021
	Post hoc intermediate		RTR ~**FT** + (1|SP) + (1|ID)	49.2	1	<0.001
	Post hoc animal based		RTR ~**FT** + (1|SP) + (1|ID)	126.8	1	<0.001
3.	Feeding trial 1		MRT ~**DIET** + (1|SP) + (1|ID)	2.5	2	0.282
	Feeding trial 2		MRT ~**DIET** + (1|SP) + (1|ID)	1.5	2	0.476
4.	MRT ~DIET + FT +**DIET:FT** + (1|SP) + (1|ID)			18.5	2	<0.001
	Post hoc plant based		MRT ~**FT** + (1|SP) + (1|ID)	20.8	1	<0.001
	Post hoc intermediate		MRT ~**FT** + (1|SP) + (1|ID)	2.3	1	0.122
	Post hoc animal based		MRT ~**FT** + (1|SP) + (1|ID)	8.9	1	0.003
5.	OL ~**DIET** + BS + (1|SEX) + (1|ORG)			0.6	2	0.742
	OM ~**DIET** + BS + (1|SEX) + (1|ORG)			5.0	2	0.083
6.	Gizzard	RTR ~**OL** + (1|DIET) + (1|SP) + (1|ID)		3.5	1	0.061
	Small intestine	RTR ~**OL** + (1|DIET) + (1|SP) + (1|ID)		1.0	1	0.322
	Gizzard	RTR ~**OM** + (1|DIET) + (1|SP) + (1|ID)		2.1	1	0.147
	Small intestine	RTR ~**OM** + (1|DIET) + (1|SP) + (1|ID)		0.9	1	0.334
	Gizzard	RTR ~**VOL** + (1|DIET) + (1|SP) + (1|ID)		2.2	1	0.134
7.	Gizzard	MRT ~**OL** + (1|DIET) + (1|SP) + (1|ID)		1.3	1	0.238
	Small intestine	MRT ~**OL** + (1|DIET) + (1|SP) + (1|ID)		4.8	1	0.029
	Gizzard	MRT ~**OM** + (1|DIET) + (1|SP) + (1|ID)		0.8	1	0.365
	Small intestine	MRT ~**OM** + (1|DIET) + (1|SP) + (1|ID)		0.0	1	0.972
	Gizzard	MRT ~**VOL** + (1|DIET) + (1|SP) + (1|ID)		1.5	1	0.216
Trial 3						
8.	Esophagus		RTR ~**SV** + (1|SP) + (1|ID)	3.0	1	0.082
	Proventriculus		RTR ~**SV** + (1|SP) + (1|ID)	9.4	1	0.002
	Gizzard		RTR ~**SV** + (1|SP) + (1|ID)	18.2	1	<0.001
	Small intestine 1st half		RTR ~**SV** + (1|SP) + (1|ID)	0.4	1	0.506
	Small intestine 2nd half		RTR ~**SV** + (1|SP) + (1|ID)	2.6	1	0.110
	Ceca		RTR ~**SV** + (1|SP) + (1|ID)	2.0	1	0.157
	Colon		RTR ~**SV** + (1|SP) + (1|ID)	2.7	1	0.099
	Feces		RTR ~**SV** + (1|SP) + (1|ID)	3.1	1	0.079

Notes

Test statistics are given for contribution of terms in bold to the presented model.

BS: body size; FT: feeding trial; ID: mallard identity; MRT: mean retention time; OL: organ length; OM: organ mass; ORG: organ; RTR: proportion retrieved; SEX: mallard sex; SP: seed species; SV: seed volume; VOL: organ volume.

The effect of diet treatment on the length and mass of digestive tract sections was tested using LMMs with diet as fixed factor, body size as covariate, and mallard sex as random effect (test 5). As a measure of body size, we used the scores of the first component of a principal components analysis on three structural size measures: tarsus, head bill, and total body length. The first principal component explained 92.1% of the variance (eigenvalue = 42.2) and correlated positively with all three size measures. First we tested for an effect of diet type on total length and mass of the digestive tract, and subsequently tested for effects on the different sections of the digestive tract.

To test the direct relations between digestive tract traits and seed gut passage irrespective of diet treatment, we used the same models as described above (binomial GLMMs for seed retrieval and LMMs for seed retention time), but with diet treatment as random effect instead of explanatory factor. Seed species and mallard ID were also included as random effects (tests 6 and 7). We tested the effect of separate digestive organ measures, which were highly correlated with each other and were therefore included as fixed effects in separate models to avoid collinearity. We used Bonferroni‐corrected significance levels to correct for multiple comparisons. Only retrieval and retention time data from feeding trial 2 were used, since organ sizes were measured shortly afterwards.

The effect of seed size on the proportion of seeds retrieved from each separate digestive organ after feeding trial 3 was also tested with binomial GLMMs with logit link function, and with seed volume as explanatory variable (test 8). Plant species and mallard ID were included as random effects. Bonferroni's correction was applied for repeated testing per digestive tract section. Data were log‐transformed when necessary to obtain normality. For all statistical analyses, we used the packages lme4 (Bates, Mächler, Bolker, & Walker, 2014) and multcomp (Hothorn, Bretz, & Westfall, 2008) in R version 3.0.3 (R Core Team, 2014).

## 3 RESULTS

### 3.1 Diet effects on seed gut passage

**Figure 2:**
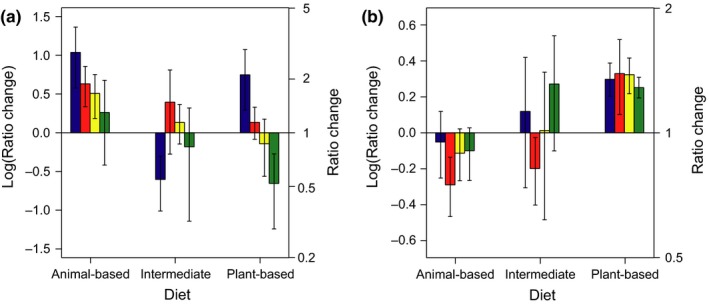
Effect of experimental diet on changes in intact gut passage and retention time of seeds. Diet treatment effects are expressed as the log‐transformed mean (± SE) ratio of post‐diet to pre‐diet total retrieval of (a) intact seeds and (b) mean retention time of seeds within individual mallards (untransformed ratio on secondary y‐axis). The four seed species tested in the feeding trials are indicated by different colors (blue = *Berula erecta*, red = *Mentha aquatica,* yellow = *Comarum paluste*, green = *Lysimachia vulgaris*). The x‐axis denotes the diet group (animal‐based, intermediate or plant‐based)

The proportion of seeds retrieved intact after adaptation to the different diets (feeding trial 2) varied significantly between diet groups (*p* = 0.035, see also Table 3), with retrieval being lowest in mallards adapted to the plant‐based diet (mean 4.3% per mallard for all four species combined, range 1.4%–8.5%), intermediate in the intermediate group (mean 6.6%, range 0.4%–17.1%), and highest in the animal‐based diet group (mean 19.8%, range 2.9%–49.4%). However, a similar pattern was observed already in the pretreatment feeding trial (trial 1), where mean seed retrieval was 3.4% (range 0.0%–7.1%) in the plant‐based group, 10.5% (range 1.3%–25.6%) in the intermediate group, and 11.8% (range 5.1%–17.8%) in the animal‐based group (significant diet effect: *p* = 0.026). Nonetheless, a strongly significant effect of the interaction between feeding trial and diet on the intact retrieval of seeds (*p* < 0.001) indicated that digestive efficiency within diet groups had not changed in the same way between feeding trials 1 and 2. Further testing within diet groups revealed that seed retrieval did not change significantly between trial 1 and trial 2 in the plant‐based group (26.5% difference, *p* = 0.021 with a Bonferroni corrected *α* = 0.017), but did decrease significantly in the intermediate group (36.8% decrease, *p* < 0.001) and increased in the animal‐based group (68.0% increase, *p* < 0.001). When focusing on the ratio of retrieval (after/before the diet treatment) between diet groups, a clear trend is visible of increased seed retrieval from mallards on animal‐based diets, and relatively low seed retrieval from mallards on intermediate and plant‐based diets (Figure 2a). *Berula erecta* deviated from the general pattern and was retrieved least in the intermediate group.

Mean retention times did not differ between the three diet groups in either of the two feeding trials (pretreatment mean: 4.4 hr ±2.2 *SD*,* p* = 0.282; post‐treatment mean: 4.3 hr ±2.4 *SD*,* p* = 0.476). The interaction between diet and feeding trial, however, did significantly affect mean retention time (*p* < 0.001). Within diet groups, mean retention time was longer after the diet treatment in the plant‐based group (1.1 hr increase, *p* < 0.001), did not change in the intermediate group (0.8 hr difference, *p* = 0.122), and was shorter in the animal‐based group (0.6 hr decrease, *p* = 0.003). Also, the mean ratio of post‐treatment to pretreatment retention times in individuals within diet groups indeed showed a trend with on average 35.6% increased retention times in the plant‐based diet group and 12.6% reduced retention times in the animal‐based diet group (Figure 2b).

### 3.2 Effect of diet on gut morphology

**Figure 3:**
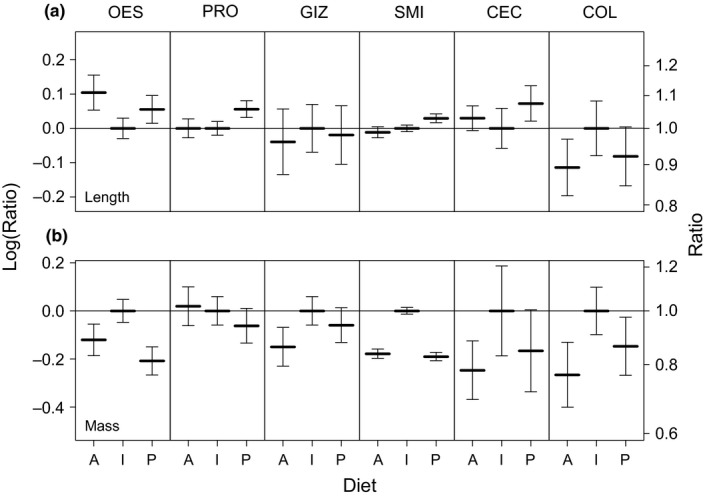
Relative length and mass of mallard digestive organs in relation to diet quality. Relative difference in (a) length and (b) mass (± *SE*) of the separate sections of the digestive tract of mallards between the three diet groups (A = animal‐based, I = intermediate, P = plant‐based). To facilitate comparison between the sections, the plant‐based and animal‐based diets are scaled relative to the median of the intermediate diet (most closely representing the pre‐diet food conditions). Whiskers denote the 5th and 95th percentiles around the median. Names of the digestive tract sections are abbreviated (OES = esophagus, PRO = proventriculus, GIZ = gizzard, SMI = small intestine, CEC = ceca, COL = colon). Diet type had no significant effect on any of the organ measures.

Digestive tract analysis revealed that differences in average length or mass of digestive organs did not follow the expected patterns. Mallards on a plant‐based diet did not have larger (*p* = 0.742) or heavier (*p* = 0.083) organs than those on an intermediate or animal‐based diet (Figure 3). Even for the organs for which we expected the strongest effects (gizzard and small intestines), we found no significant diet effect (gizzard volume: *p* = 0.299; small intestine length: *p* = 0.747). On average, gizzards were smallest (17.8 cm^3^ ± 4.6 *SD*) and lightest (18.4 g ± 4.7 *SD*) in the animal‐based diet group, but largest (20.3 cm^3^ ± 3.9 *SD*) and heaviest (21.4 ± 3.7 *SD*) in the intermediate group rather than in the plant‐based diet group. Small intestine length did follow the expected trend with highest values in the plant‐based group (120.5 cm ±12.0 *SD*) and lowest values in the animal‐based group (115.7 cm ±10.8 *SD*), but again, small intestines were heaviest in the intermediate group (9.8 g ± 3.3 *SD*). However, none of these differences were statistically significant (Table 3).

### 3.3 Effect of gut morphology on seed gut passage

Variation in digestive tract morphology between individual mallards within and between diet groups was high, with individual gizzard mass for example ranging from 12.9 to 26.1 g and small intestine length ranging from 91 to 137 cm. Nonetheless, we found no direct relation between total digestive tract length or mass and the proportion of seeds retrieved in the post‐diet feeding trial 2 (*p* = 0.758 and *p* = 0.174, respectively). Also none of the other organ properties was related to intact gut passage. Similarly, length and mass of the total digestive tract were unrelated with seed retention time (*p* = 0.59 for both measures). Mean seed retention time was positively related to small intestine length (*p* = 0.029) and colon length (*p* = 0.018), but these relations were no longer significant after Bonferroni's correction. Hence, we found no conclusive relations between digestive tract morphology and seed gut passage time or intact passage.

### 3.4 Seed size and retention patterns

**Figure 4:**
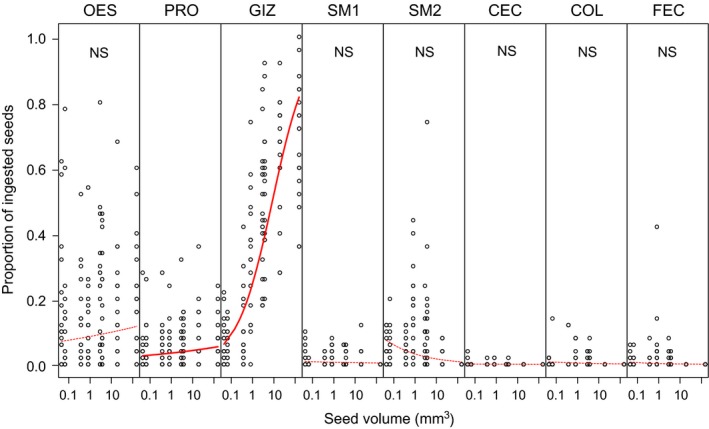
Relation between seed volume and position in the digestive tract 3 hr after feeding. Distribution of ingested seeds over the separate parts of the digestive tract, and the feces, as measured 3 hr after feeding. Note that the x‐axis is on a log scale. Names of the digestive tract sections are abbreviated (OES = esophagus, PRO = proventriculus, GIZ = gizzard, SM1 = first half of small intestine, SM2 = second half of small intestine, CEC = ceca, COL = colon, FEC = feces). Thick solid fitted lines denote the significant relations, whereas nonsignificant trends are shown as dotted lines

Digestive tract analysis three hours after feeding the mallards with differently sized seeds (feeding trial 3) revealed large differences in retention between seeds of different sizes. Of the largest two species, *Iris pseudacorus* and *Sparganium erectum*, 96.4% and 94.2% of the ingested seeds, respectively, were still present inside the digestive tract. In contrast, only 29.6% and 32.2% of the two smallest species, *Epilobium palustre* and *Hypericum tetrapterum,* were still present. This size effect was strongest in the upper parts of the digestive tract (Figure 4), where most seeds were retained (50% of all seeds were retrieved from the gizzard). There was a significantly positive relation between seed size and the proportion of seeds present in the proventriculus (*p* = 0.002) and especially in the gizzard (*p* < 0.001). Conversely, in digestive tract sections beyond the gizzard, there was a negative trend between seed size and the proportion of seeds, albeit not significant in any of the organs. The total number of seeds in the second half of the small intestines exceeded the number of seeds in the first half. Very few seeds were retrieved from the ceca and colon, and we found no clear relation with seed size in those parts. We found no evidence that heavier gizzards or small intestines contained more seeds (LM: *R* ^2^ = 0.06, *p* = 0.313 and *R* ^2^ = 0.01, *p* = 0.668, respectively).

## 4 DISCUSSION

In this study, we aimed at gaining mechanistic insights in the effects of diet quality and seed size on seed gut passage by means of three feeding trials. The results of the feeding trials demonstrated in the first place that individual mallards show large variability in digestive parameters. This makes it challenging to detect clear effects of diet quality on seed dispersal potential. Still, we found a general pattern consistent with our hypothesis based on earlier studies (Charalambidou et al., 2005; Kehoe et al., 1988; Miller, 1975) that the proportion of seeds passing intact increased and retention times became shorter with a larger proportion of animal‐based diet content. This supports the hypothesis that seeds have higher potential to survive gut passage when consumed by mallards on an animal‐based diet, but may be dispersed over shorter distances than those consumed by mallards on a plant‐based diet (Charalambidou et al., 2005).

By performing postmortem digestive tract analysis, we expected to find that this difference in digestive parameters is directly mediated by different digestive organ sizes between diet groups (Kehoe et al., 1988; Miller, 1975). However, sizes of digestive tract sections did not show any clear differences between diet treatments. The experimental setup did not enable comparison between pre‐ and post‐diet digestive tract traits (and thus examination of changes in digestive organ size within individuals), so it is still possible that morphology had changed but remained undetected. This could explain why mean seed retention time did not vary between diet groups, but had clearly changed within treatment groups during the diet experiment. Regardless of diet treatment, we found a weak indication that longer retention times are associated with long intestines, although the results of feeding trial 3 could not confirm that longer guts contain more seeds. Feeding trial 3 did show that, three hours after ingestion, large seeds were present in larger numbers in the upper digestive tract than small seeds. Combined with the inverse trend in the small intestine, this suggests that large seeds are retained mostly in the gizzard, the organ where mechanical digestion occurs and seeds are destroyed (Kleyheeg, 2015).

### 4.1 Effect of diet on seed retrieval

To optimize energy uptake, mallards and other vertebrates may adjust the morphology of their digestive tract when diet quality changes (Karasov & Carey, 1996; Kehoe et al., 1988; Miller, 1975; Oudman et al., 2016; Starck, 2003). Accordingly, this should affect the efficiency of digesting food particles, including seeds. Indeed, mallards on an animal‐based diet showed a significantly higher retrieval of seeds than both other diet groups, similar to results of Charalambidou et al. (2005). However, already before the diet treatment there was a significant difference between the animal‐based and plant‐based diet group. Therefore, we specifically examined the change in digestive parameters within diet groups before and after the diet treatment. In accordance with our hypothesis, after four weeks of adjustment to different diets, the digestive efficiency in birds on an animal‐based diet had slightly decreased (i.e., more seeds were retrieved intact), while little change had occurred in the digestive efficiency in the intermediate or plant‐based treatment. This pattern was clear for all seed species but *B. erecta*, which showed an unexplained reduced intact gut passage in mallards on an intermediate diet. Although the differences between diet groups were not quite as strong as observed by Charalambidou et al. (2005), these results support their conclusion that dispersal potential for seeds depends on the general diet of a mallard. Mean retention time did not differ between diet groups, but again we did observe differences in changes within diet groups. Mean retention time was especially increased in the plant‐based diet group, while remaining highly variable in the intermediate group and changing only a little in the animal‐based diet group. Charalambidou et al. (2005) already detected changes in seed retention time for indigestible plastic markers, but this study is the first to indicate that digestible plant seeds are also retained longer in mallards on a plant‐based diet. Mallards shift from a primarily animal‐based diet in spring and early summer to a seed‐based (high fiber) diet in autumn and winter (Dessborn et al., 2011). This suggests that seed digestion is relatively high, but seed retention time relatively long, during autumn–winter. In this period, migration and increased regional movements of waterfowl coincide with a shift toward a more seed‐based diet, increasing the probability for rare, but long‐distance dispersal events (Kleyheeg et al., 2017; Nathan et al., 2008; Viana et al., 2013). Since digestive parameters may differ significantly between inactive captive birds and active wild birds (Kleyheeg et al., 2015; Leeuwen, Tollenaar, et al., 2012), absolute dispersal values reported here should be treated with care when making inferences about the field situation.

### 4.2 Causes and consequences of digestive organ size

Plant‐based, high‐fiber food items are relatively hard to digest. Miller (1975) and Kehoe et al. (1988) observed that mallards respond to a shift towards a high‐fiber diet by enlarging their digestive organs, as shown in gizzard mass and in both mass and length of the small intestine, ceca, and colon. However, despite the relatively long adjustment period of four weeks in our study, we found no significant difference in the size of any digestive tract part between the diet groups in this study. Since we examined the digestive tract sections through carcass analysis, we were unable to observe within‐individual changes, which might have been much more substantial than we were capable of detecting by our approach.

When combining the observations of digestive tract adaptation (Kehoe et al., 1988; Miller, 1975) and seed retrieval (Charalambidou et al., 2005) in response to diet quality, one would expect a causal relationship, namely that more seeds are destroyed by larger digestive organs developed in adaptation to a plant‐based diet. However, none of the size measures of the digestive tract contributed significantly to the variation in seed retrieval or retention time. This indicates that digestive efficiency, and thus seed retrieval, is not only determined by weight and length of the various digestive organs, and that other aspects, such as muscle and enzyme activity, must play a role. The large variation in seed retrieval between individuals feeding on a similar diet does suggest opportunities for seed dispersal in the field throughout the annual cycle.

### 4.3 Seed size and retention time

The relation between seed size and gut passage time has often been described, but the direction of this relation differs between studies (e.g., positive in Soons et al. (2008), while negative in Kleyheeg et al. (2015)). Experiments to test the underlying mechanisms are lacking. In feeding trial 3 described here, we show that passage of larger seeds is delayed in multiple digestive tract sections, but mostly in the upper part. Firstly, large seeds tended to be overrepresented in the esophagus, which contains the crop, which has a storage function of food before it passes to the organs where most digestion occurs (Ziswiler & Farner, 1972). Secondly, relatively high numbers of larger seeds were found in the proventriculus, which is a short organ where seeds are probably cued and pretreated with gastric juice before they enter the gizzard. Finally, the strongest seed size effect on retention was observed in the gizzard, from which most large seeds were retrieved three hours after seed ingestion. Small seeds may exit the gizzard more likely by chance through the relatively narrow pylorus, which connects the gizzard with the small intestine. An alternative, but not mutually exclusive, explanation is that species‐specific differences in abundance in the gizzard are caused by more efficient digestion of small seeds. This would be in agreement with earlier findings that large seeds require more force to be crushed in the gizzard than small seeds (Kleyheeg, 2015; Reynolds & Cumming, 2016). However, in the small intestine, the relation between seed size and relative abundance is gone or even negative, providing evidence that small seeds do pass the gizzard more quickly. The retention of large seeds in the proventriculus and gizzard has consequences for their survival. In the proventriculus hard food particles like seeds are pretreated with gastric juice to aid mechanical digestion, which occurs in the gizzard (Ziswiler et al., 1972). The probability of destruction increases with time in both organs, and hence, the prolonged retention in this part of the digestive tract is a likely mechanism underlying the often observed negative relation between seed size and gut passage survival (e.g., Soons et al., 2008; Mueller & Valk, 2002). Although counterintuitive, the delayed passage through the gizzard can explain both the positive and the negative relation between seed size and retention time found in different studies. For resilient large seeds, which survive mechanical treatment in the gizzard, the retention in the gizzard eventually results in longer gut passage times than for small seeds. On the other hand, soft large seeds (e.g., those used in Kleyheeg et al., 2015) will always be destroyed with prolonged retention in the gizzard and only the few seeds that do pass rapidly will be excreted intact, resulting in short retention times. Differences in resilience in similarly sized seeds may be caused by a variety of other seed traits, including shape, seed coat thickness, seed coat permeability, seed surface smoothness, and fiber content (Kleyheeg, 2015; Kreitschitz, Kovalev, & Gorb, 2016; Mueller & Valk, 2002; Soons et al., 2016; Wongsriphuek et al., 2008). Hence, the gizzard and seed resilience together modulate the retention time of large seeds.

## 5 CONCLUSIONS

This study is the first to test within individual waterfowl how diet adaptation and variation in digestive organ size may affect the dispersal potential of ingested plant seeds. After four weeks of adaptation to different diets, we found no consistent differences in digestive organ size between diet groups, suggesting that digestive organ size variation is naturally high, even within birds feeding on the same food type. Nonetheless, digestion of seeds within individual mallards was reduced after adjustment to a more animal‐based diet. This suggests that adaptation to digestion of food of different quality may already be achieved by small changes in digestive organ size, or that other physiological mechanisms play a (potentially additive) role. Either way, the rapid adjustment to diet shifts enables mallards to cope with variable and unpredictable environmental conditions, which has consequences for seed survival of digestive tract passage. Mallards adapting to a plant‐based diet showed an increase in seed retention time, although we could not show a direct relation with digestive organ size. Regardless of diet type, prolonged retention of seeds in the gizzard is seed size related, which in the first place provides time for pretreatment with gastric juices to soften the seeds, and meanwhile increases the probability that the seeds break down (Kleyheeg, 2015). This prolonged exposure to chemical and mechanical digestion underlies the negative relation between seed size and intact gut passage, as well as the lower viability of large seeds that are excreted intact (Soons et al., 2008). Hence, the interplay between digestive processes and seed resilience finally determines the retention time and survival of large seeds. These results improve our mechanistic understanding of the regulation of dispersal potential of plant seeds by waterfowl in naturally variable ecosystems.

## AUTHOR CONTRIBUTIONS

EK conceived the study and all authors contributed significantly to the experimental design. EK and SO collected the data. All authors wrote the manuscript together and approved the final version.

## DATA ACCESSIBILITY

The data presented in this manuscript are available from https://datadryad.org under https://doi.org/10.5061/dryad.nt7s243.
